# Alteration of renal Na,K‐ATPase in rats following the mediastinal γ‐irradiation

**DOI:** 10.14814/phy2.13969

**Published:** 2019-02-11

**Authors:** Barbora Kaločayová, Ivona Kovačičová, Jana Radošinská, Ľubomíra Tóthová, Lucia Jagmaševič‐Mézešová, Marko Fülöp, Ján Slezák, Pavel Babál, Pavol Janega, Norbert Vrbjar

**Affiliations:** ^1^ Centre of Experimental Medicine Institute for Heart Research Slovak Academy of Sciences Bratislava Slovak Republic; ^2^ Institute of Physiology Faculty of Medicine Comenius University in Bratislava Bratislava Slovak Republic; ^3^ Institute of Molecular Biomedicine Faculty of Medicine Comenius University Bratislava Slovak Republic; ^4^ Slovak Medical University Bratislava Slovak Republic; ^5^ Institute of Pathology Faculty of Medicine Comenius University in Bratislava Bratislava Slovak Republic

**Keywords:** Abscopal effect, enzyme expression, enzyme kinetics, sodium pump

## Abstract

Na,K‐ATPase represents the key enzyme that maintains the homeostasis of sodium and potassium ions in the cells. It was documented that in directly irradiated organs the activity of this enzyme is decreased. The aim of present study was to clarify the remote effect of irradiation in mediastinal area on the activity of the Na,K‐ATPase in kidneys in rats. Ionizing radiation in single dose 25 Gy resulted in consequent decrease of the body weight gain as well as the size of kidneys in Wistar rats. In addition, radiation induced alterations in the oxidative status of blood plasma. Irradiation also decreased the activity of renal Na,K‐ATPase. Measurements of enzyme kinetics that were dependent on the concentration of energy substrate ATP or cofactor Na^+^ indicated that the lowered enzyme activity is probably a consequence of decreased number of active molecules of the enzyme, as suggested by lowered *V*_max_ values. Immunoblot analysis confirmed the lowered expression of the catalytic alpha subunit together with decreased content of the glycosylated form of beta subunit in the renal tissue of irradiated rats. The ability of the enzyme to bind the substrate ATP, as well as Na^+^ was not affected, as shown by unaltered values of *K*_m_ and K_N__a_. Irradiation of the body in the mediastinal area despite protection of kidneys by lead plates during application of X‐ray was followed by significant decline of activity of the renal Na,K‐ATPase, what may result in deteriorated homeostasis in the organism.

## Introduction

Radiation therapy is commonly used therapeutic procedure in oncology. The effects of radiation therapy are mediated, in addition to their direct impact on DNA, by the production of free radicals. Proper functioning of membranes is unavoidable for biological systems, and their integrity is essential for normal cell functions. Radiation‐induced increase in free radicals results in lipid peroxidation, leading to structural and functional damage to cellular membranes (Purohit et al. 1980). The damage to membrane organization is an initial step in cell death. During radiotherapy, formation of hydroperoxides in membranes would result in the damage of membrane‐bound enzymes. One of these enzymes is the Na,K‐ATPase or so called sodium pump. In all living cells, this enzyme keeps the intracellular balance of Na^+^ and K^+^ by transporting three ions of Na^+^ out of the cell in exchange for two ions of K^+^ into the cell utilizing the energy from ATP hydrolysis. In our previous study, using enzyme kinetic measurements of the cardiac Na,K‐ATPase in 20‐weeks‐old male rats, we observed impairment in the affinity of the Na^+^‐binding site together with decreased number of active Na,K‐ATPase molecules 6 weeks after mediastinal gamma‐irradiation at a dose of 25 Gy. These changes most probably result in deteriorated efflux of the excessive Na^+^ from the intracellular space in hearts of irradiated rats (Mézešová et al. 2014). This enzyme having a key role in maintaining the cellular homeostasis of sodium ions throughout the organism was affected by irradiation in various other organs like intestine (Lebrun et al. 1998), kidney (Balabanli et al. 2006) and erythrocytes (Moreira et al. 2008; Chitra and Shyamaladevi 2011). The Na,K‐ATPase represents the main consumer of intracellularly produced energy in the renal tissue (Welch 2006). It indicates the possible importance of the enzyme complications induced by radiotherapy. Our study was oriented to the investigation of probable indirect abscopal effect (a phenomenon where the response to radiation is seen in an organ/site distant to the irradiated area) of mediastinal irradiation of rats on functional properties of the enzyme in kidney. This organ is highly sensitive to radiation‐induced late effects resulting in the so called radiation nephropathy which occurred dramatically during second to sixth weeks after local direct irradiation of porcine kidneys (Cohen et al. 2000; Cohen and Robbins 2003). However, there is still lack of information concerning the molecular mechanism of Na,K‐ATPase disturbances in the above tissue after irradiation. Investigations of the enzyme properties in such condition bring new insight into the processes involved in maintenance of sodium homeostasis in kidney after radiotherapy.

## Materials and Methods

### Animal model and radiation

**Figure 1:**
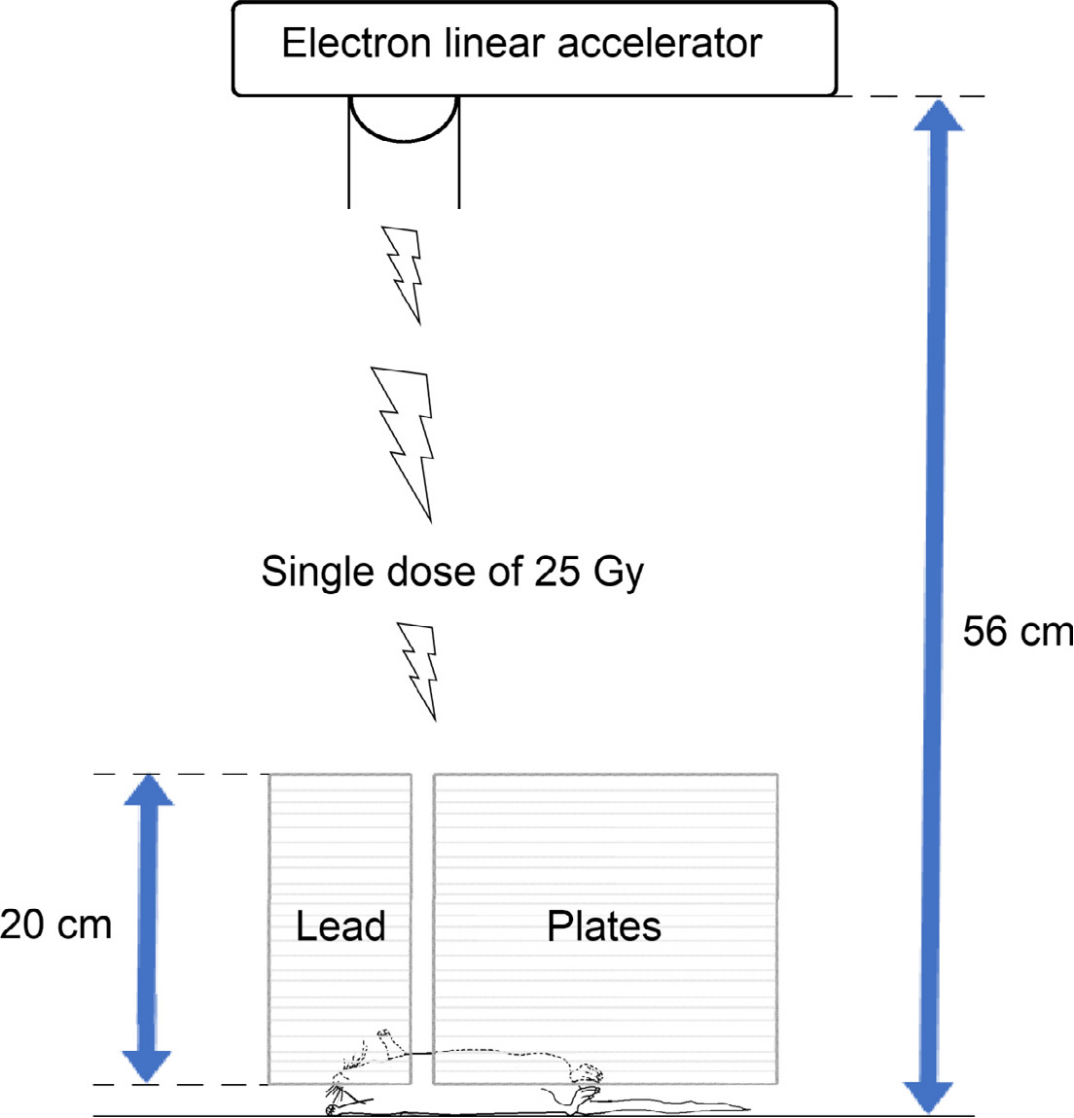
Schematic presentation of the irradiation procedure of rats. Rats were irradiated by a single dose of 25 Gy in the mediastinal area using experimental chamber where the rest of the body was protected by 20 cm thick lead plates.

All procedures in this study were approved by the Institutional Animal Care Committee. Male Wistar rats were obtained from Velaz Praha (Czech Republic) and maintained in our animal care facility on a 12:12‐h light/dark cycle with free access to food and water. At the age of 14 weeks, animals (*n* = 12) were anesthetized with thiopental (65 mg·kg^−1^ b.w.). Rats were irradiated with 5 MeV/1 kW electron linear accelerator UELR 5‐1S with tungsten converter to X‐rays at the dose rate 10 Gy/min (Producer NIIEFA St. Petersburg, RF). A single dose of 25 Gy was given locally on mediastinal area. Irradiation was directed transversally, crossing the chest of the rat at the heart level while the rest of the animal was shielded with 20 cm thick lead plates (Fig. 1). A single dose of 25 Gy to the heart corresponds to the cumulative dose of irradiation commonly used in patients. Six weeks after irradiation the rats were anesthetized with thiopental (65 mg·kg^−1^ b.w.) and sacrificed by heart excision. Age‐matched Wistar male rats from the same breeding facility served as controls (*n* = 12). After excision of the heart, blood from the chest cavity was immediately collected. Sodium‐salt heparin was used as anticoagulant. Kidneys were quickly removed, rapidly rinsed with ice‐cold physiological saline, weighed, frozen in liquid nitrogen, and stored until use.

### Biochemical analysis of oxidative status

Markers of protein oxidation were measured by spectrophotometric analysis of advanced oxidation protein products (AOPP) (Witko‐Sarsat et al. 1996). Briefly, plasma samples (diluted 1:4 in phosphate‐buffered saline (PBS), pH = 7.2) were mixed with glacial acetic acid. For the calibration curve construction, chloramine T with potassium iodide was used. The absorbance was measured at 340 nm.

The markers of carbonyl stress – advanced glycation end products (AGEs) and fructosamine were determined. AGEs were measured spectrofluorometrically. Samples were diluted with PBS in the ratio 1:4. Fluorescence was measured at λex = 370 nm and λem = 440 nm (Münch et al. 1997). For fructosamine measurement, 20 *μ*L of samples and standards (1‐deoxy‐morpholino‐d‐fructose) were added to the microtiter plate. Thereafter, nitro blue tetrazolium was added, and the reaction was shortly mixed and incubated at 37°C for 15 min. The absorbance was measured at 530 nm (San‐Gil et al. 1985).

The following markers of antioxidant status were measured: total antioxidant capacity (TAC) and ferric reducing antioxidant power (FRAP). For TAC assessment, the plasma was mixed with acetate buffer (pH = 5.8). The initial absorbance was measured at 660 nm as blank. When ABTS solution (2.2′‐azino‐bis(3‐ethylbenzothiazoline‐6‐sulfonic acid with acetate buffer) was added, the absorbance (660 nm) was measured again. FRAP was measured after the addition of FRAP reagent (warmed to 37°C, composed of acetate buffer (pH = 3.6), tripyridyl‐s‐triazine, FeCl_3_.6H_2_O, and water) to the microtiter plate. Afterward, initial absorbance was measured as blank. The samples were added to the reagent and measured again at 593 nm (Benzie and Strain 1996).

The concentration of proteins was measured by bicinchoninic acid kit (Sigma‐Aldrich, Munich, Germany), accord**i**ng to the manufacturer's instructions. The bovine serum albumin was used as standard. All measurements were performed on a Tecan Sapphire II Instrument (Grödig, Austria) and reagents used for these measurements were obtained from Sigma‐Aldrich (Munich, Germany).

### Evaluation of markers of kidney function

Biochemical markers (creatinine and urea) were measured at certified biochemical laboratory (Medirex, University Hospital Ružinov, Bratislava, Slovakia) using analyzer Olympus AU400 (Beckman Coulter, California, USA).

### Histology of kidney

Kidney tissue was fixed in 10% formalin and routinely processed in paraffin. Histological sections were stained with hematoxylin and eosin and with phosphotungstic acid hematoxylin. The change in the size of the kidney glomeruli was morphometrically evaluated in slides stained with hematoxylin end eosin by measuring the cross‐section area in pixel number with ImageJ 1.42q program (National Institute of Health, USA). The results were statistically analyzed by GraphPad Prism (GraphPad Software, USA). From each kidney several slices were analyzed amounting to more than 80 in controls and to more than 120 in irradiated group.

Phosphotungstic acid hematoxylin stain that identifies basic proteins was used for identification of mitochondrial matrix proteins (Silverman and Glick 1969). Blue‐stained grains in the cytoplasm of proximal tubules epithelium were isolated as an area by digital color extraction and morphometrically evaluated by the same program.

### Assay of Na,K‐ATPase activity

The plasmalemmal membrane fraction from kidney was isolated according to (Jorgensen 1974). Amount of protein was determined by the procedure of (Lowry et al. 1951) using bovine serum albumin as a standard.

All enzyme assays were carried out at 37 °C using 10 *μ*g·mL^−1^ of membrane protein. The Na,K‐ATPase activity was estimated in an assay buffer containing (in mmol·L^−1^): 4 MgCl_2_, 100 NaCl, 10 KCl, and 50 TRIS (pH = 7.4). Subsequently, after 20 min of preincubation in substrate‐free medium, the enzyme reaction was initiated by adding increasing amount of TRIS‐ATP in the range of 0.16–8.00 mmol·L^−1^. The reaction was stopped after 20 min by adding 12% ice‐cold trichloracetic acid. The liberated inorganic phosphorus originating from ATP hydrolysis was estimated according to the method of (Taussky and Shorr 1953). In order to establish the Na,K‐ATPase activity, the ATP hydrolysis that occurred in the presence of Mg^2+^ only was subtracted. The enzyme kinetics for sodium activation was determined by the same way. The concentration of NaCl varied in the range of 2–100 mmol·L^−1^ and the amount of ATP was constant (8 mmol·L^−1^). The kinetic parameters were evaluated by direct nonlinear regression of the obtained data.

### Preparation of tissue fractions for electrophoresis and immunochemical western blot analysis

The tissue samples from kidneys were resuspended in ice‐cold buffer containing (in mmol·L^−1^): 50 Tris‐HCl, 250 sucrose, 1.0 dithiothreitol, 1.0 phenylmethylsulfonylfluoride (pH 7.4) and homogenized with a glass–teflon homogenizer. The homogenates were centrifuged at 800 g for 5 min at 4°C; pellets obtained after this centrifugation were discarded and the supernatants were centrifuged again at 9300*g* for 30 min. Following the second centrifugation, the pellets were resuspended in homogenizing buffer supplemented with 0.2% Triton X‐100 and centrifuged at 9300*g* for 1 min. The Triton X‐100‐soluble supernatants represented the particular fractions. The protein concentrations were estimated by the method of (Bradford 1976).

### Electrophoresis and immunochemical western blot analysis

Samples of particular protein fractions (for *α*1 and *β*1 Na,K‐ATPase subunit detection) containing equivalent amounts of proteins per lane (20 *μ*g per lane) were separated by sodium dodecyl sulfate‐polyacrylamide gel (12%) electrophoresis (SDS‐PAGE). For western blot assays separated proteins were transferred from gel to a nitrocellulose membrane overnight at 4°C. The quality of the transfer was controlled by Ponceau S staining of nitrocellulose membranes after the transfer. Specific antibodies against *α*1 (mouse monoclonal antibody from Sigma; product number A‐277, in dilution 1:250) and *β*1 (mouse monoclonal antibody from Santa Cruz; C464.8: sc‐21713, in dilution 1:200) subunits of Na,K‐ATPase were used for the primary immunodetection. Peroxidase‐labeled anti‐mouse (from Cell Signaling; #7076, in dilution 1:1000) immunoglobulin was used as the secondary antibody. Bound antibodies were detected by the enhanced chemiluminescence detection method (Amersham Imager 600). Densitometrical quantification of protein levels was performed by comparison to loading control beta‐actin (mouse monoclonal antibody [AC‐15] from Abcam; ab6276, in dilution 1:1000 and corresponding anti‐mouse secondary antibody) and using an ImageJ program.

### Statistical analysis

All investigated parameters are expressed as means median, 10th, 25th, 75th, and 90th percentiles as vertical boxes with error bars. Mann–Whitney Rank test were used for statistical analysis. The differences were considered to be significant when the *P*‐value was less than 0.05.

## Results

### Health condition of animals

At the end of the experiment, the irradiated animals showed significantly lower (by 28%) body weight as compared with control rats. The importance of pathophysiological complications in our experiment was emphasized also by lower body weight gain in the irradiated group compared with control and also by mortality amounting 17% in the group of irradiated animals. The kidney weight was also proportionally decreased, resulting in similar kidney weight/body weight ratio in both groups (Table 1).

**Table 1:** Weight parameters of control and irradiated (single dose 25 Gy) rats

	Bw [g] Start	Bw [g] end	Bw gain [g]	Kw [mg]	Kw/Bw [mg/g]
Controls	264 ± 15	400 ± 36	136 ± 30	2438 ± 214	6.11 ± 0.38
Irradiated	257 ± 13	290 ± 22^a^	31 ± 27^a^	1860 ± 256^a^	6.10 ± 0.29

Body weight (Bw), was estimated at the beginning and at the end of 6 weeks lasting experiment. Kidney weight (Kw) was estimated at the end of the experiment. Kw/Bw represents the ratio of the kidney weight versus body weight. Statistical significance: a ~*P* < 0.001 as compared to the control group. Data represent means ± SD, *n* = 8 in control *n* = 8 in irradiated group.

### Plasma protein concentration and biochemical analysis of oxidative status

**Figure 2:**
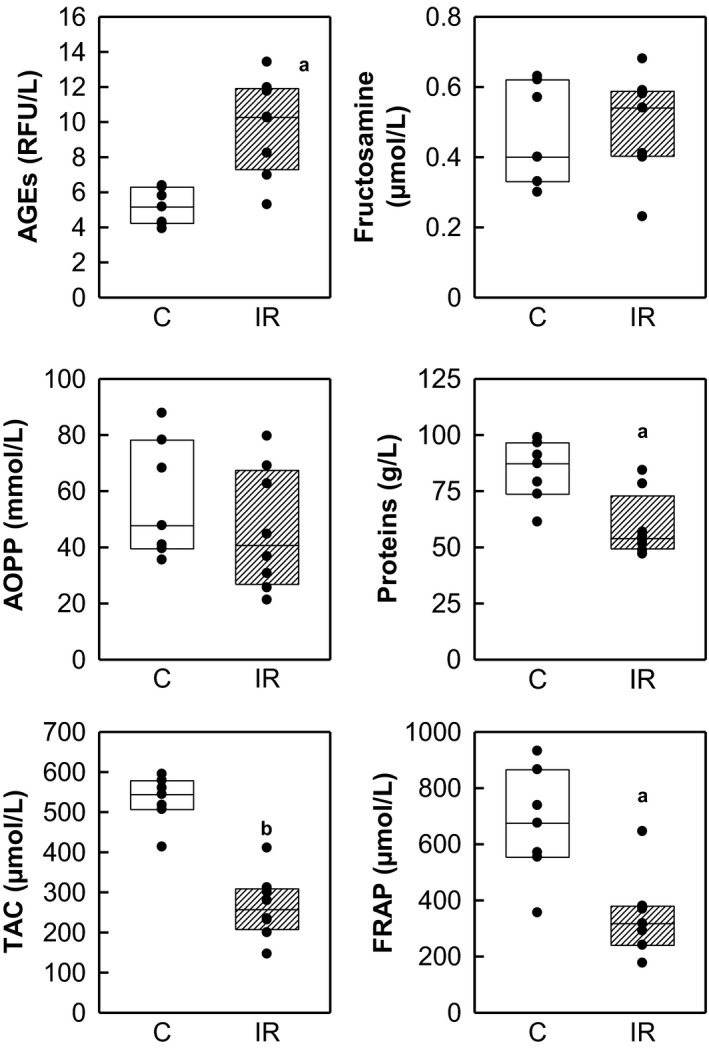
Plasma protein concentration and biochemical analysis of oxidative status in blood plasma. Concentrations of: advanced glycation end products (AGEs, RFU – relative fluorescence unit), fructosamine, advanced oxidation protein products (AOPP), total antioxidant capacity (TAC), and ferric reducing antioxidant power (FRAP). Statistical significance: a ∼ *P* < 0.005, b ∼ *P* < 0.001.

Total protein concentration in plasma was lower in irradiated rats compared with control (84 ± 5 control vs. 59 ± 5 irradiated, in g/L, *P* = 0.004). We observed statistically significant differences between the control and the irradiated group in the following parameters of oxidative stress and antioxidant status in plasma: TAC (530 ± 23 control vs. 264 ± 28 irradiated, in *μ*mol/L, *P* < 0.0001), FRAP (670 ± 75 control vs. 345 ± 57 irradiated, in *μ*mol/L, *P *= 0.005), and AGEs (5.15 ± 0.38 control vs. 9.77 ± 0.97 irradiated, in relative fluorescence units/L, *P *= 0.001). The experimental groups did not significantly differ in fructosamine plasma levels (0.46 ± 0.05 control vs. 0.49 ± 0.05 irradiated, in *μ*mol/L, *P *= 0.66) and AOPP (56.8 ± 7.9 control vs. 46.3 ± 7.7 irradiated, in mmol/L, *P *= 0.36). All the results are summarized in the (Fig. 2).

### Markers of kidney function

Creatinine and urea concentrations in plasma samples were not affected significantly by irradiation of rats in mediastinal area. The concentration of creatinine slightly decreased from 48.4 ± 8.9 to 40.6 ± 6.1 *μ*mol/L, but this alteration was not statistically significant. Concentration of blood urea nitrogen was similar in both experimental groups amounting 7.4 ± 1.2 mmol/L in controls and 7.7 ± 0.7 in irradiated rats.

### Histology of kidneys

**Figure 3:**
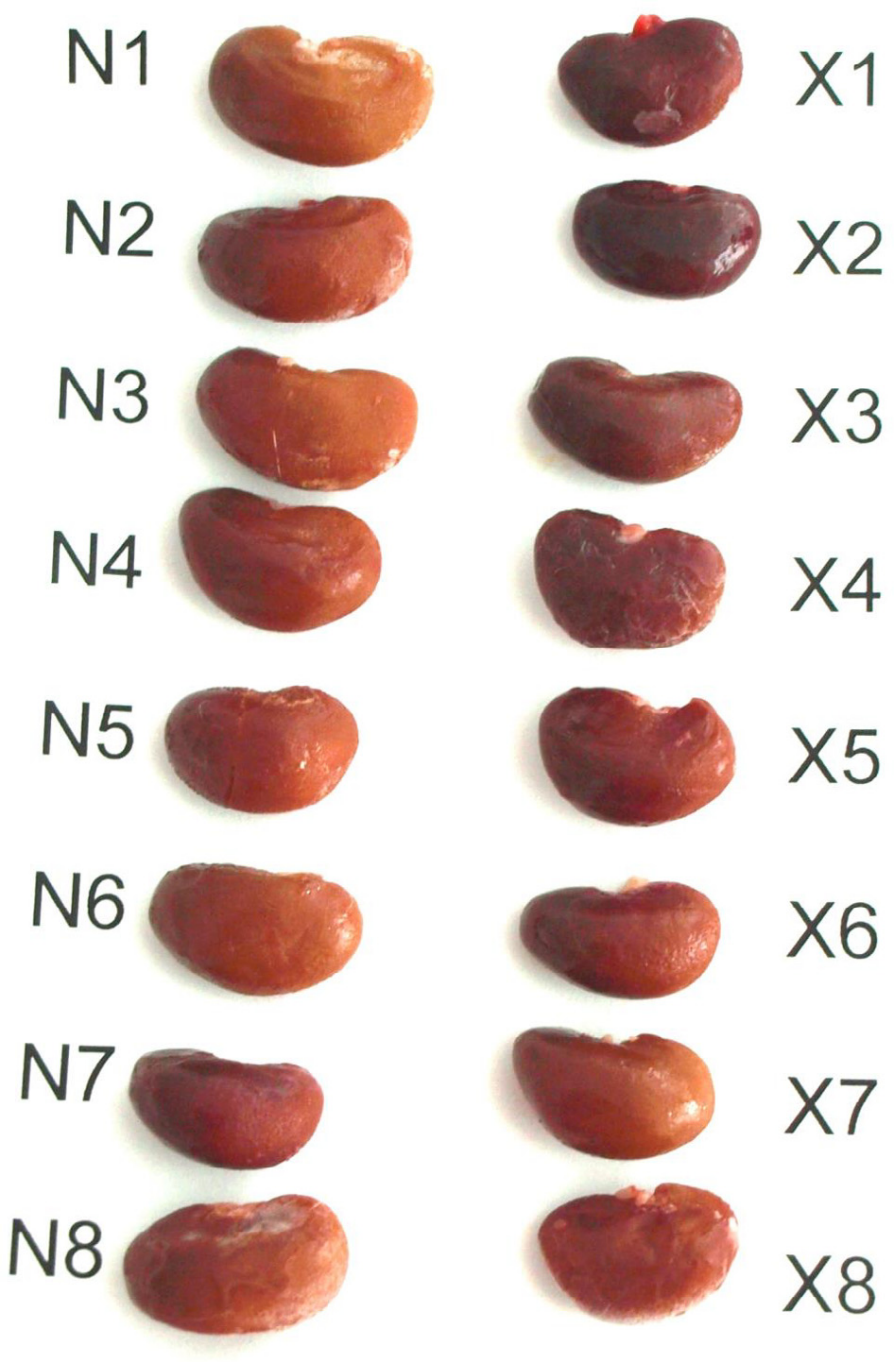
Representative photograph of kidneys of normal nonirradiated rats (N1–8) and rats irradiated by X‐ray (X1–8) in a single dose of 25 Gy.

**Figure 4:**
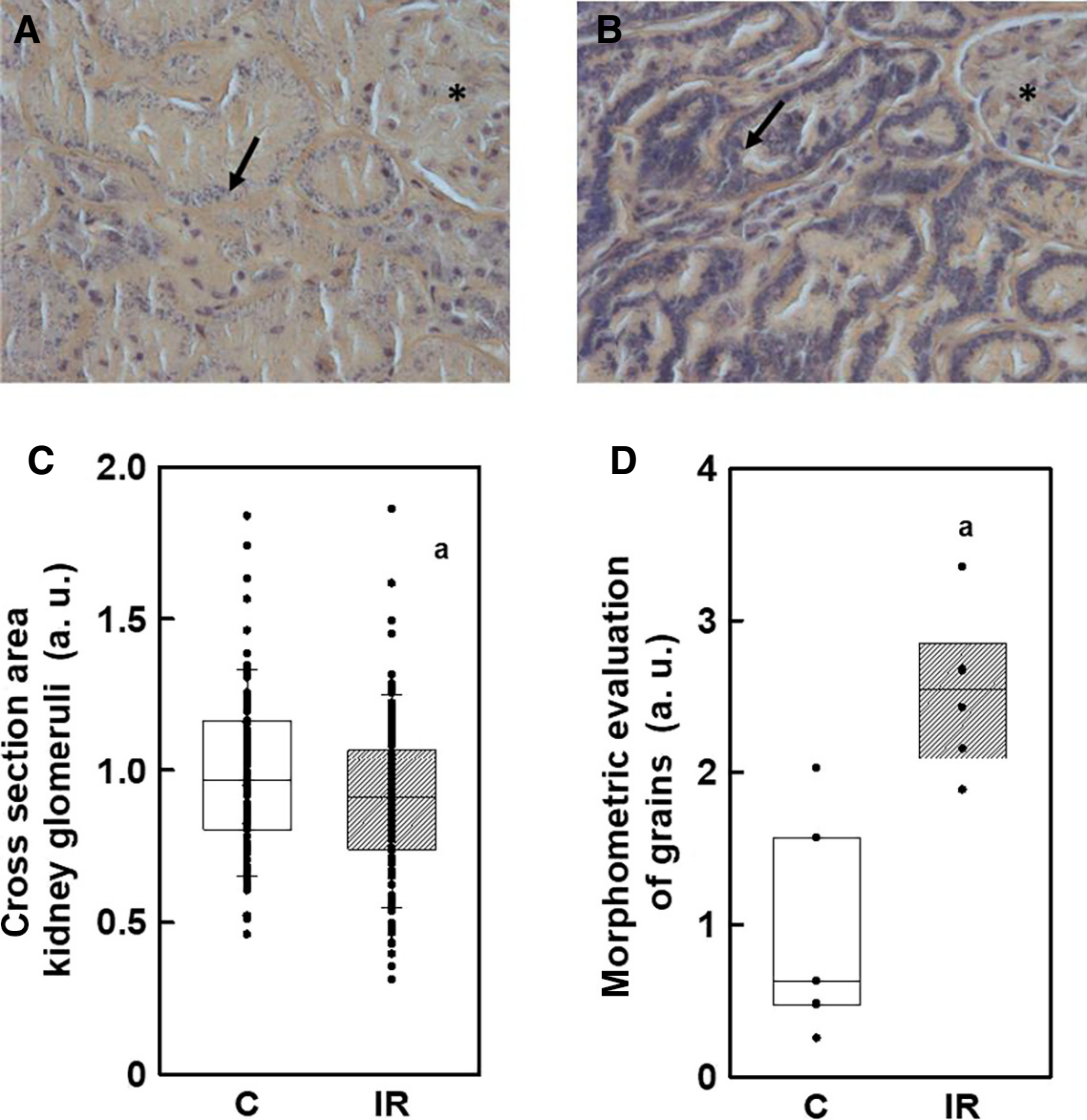
Phosphotungstic acid hematoxylin staining used for identification of mitochondrial matrix proteins. Kidney tissue with normal distribution of blue‐stained grains in the cytoplasm of proximal tubules epithelium (arrow) in control animals (A) and significantly increased density of grains in irradiated animals (B); glomerulus (*). Magnification: 400×. Evaluation of cross‐section area of kidney glomeruli in control (C) and irradiated animals (IR); *P *= 0.015 (C). Morphometric evaluation of grains representing mitochondria in the cytoplasm of proximal tubules epithelium *P* = 0.001 (D). Points in C represent individual data and D they represent means of 6–8 measurements.

The visual comparison of kidneys from irradiated rats compared with those from control rats already indicated some alterations. The kidneys from irradiated rats were smaller and ruddier (Fig. 3). Indeed, the kidney weight of irradiated rats was lowered by 21% but the kidney weight/body weight ratio was similar in both experimental groups (Table 1) indicating that the observed alteration does not represent any hypotrophy, but it is simply a consequence of lowered body weight gain. Kidney tissue in histological slides stained with routine hematoxylin and eosin did not show any remarkable morphological differences. Morphometric measurement revealed a significant decrease in the size of glomeruli in the irradiated animals (Fig. 4). Staining with phosphotungstic acid hematoxylin revealed a conspicuously increased density of blue‐stained tiny grains representing mitochondria in the cytoplasm of epithelial cells of proximal tubules in the kidneys of irradiated animals (Fig. 4). This increase proved to be highly significant (Fig. 4).

### Kinetic measurements of Na,K‐ATPase activity

**Figure 5:**
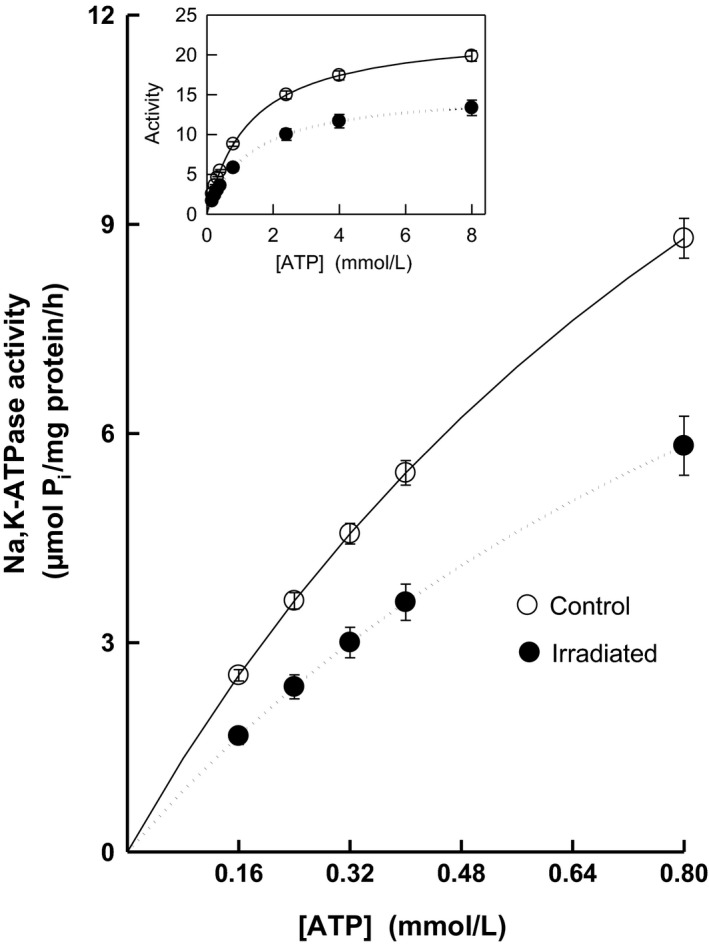
Activation of renal Na,K‐ATPase by low concentrations of substrate ATP in control and irradiated (25 Gy) Wistar rats. Insert: Activation of the enzyme in the whole investigated concentration range of ATP.

**Figure 6:**
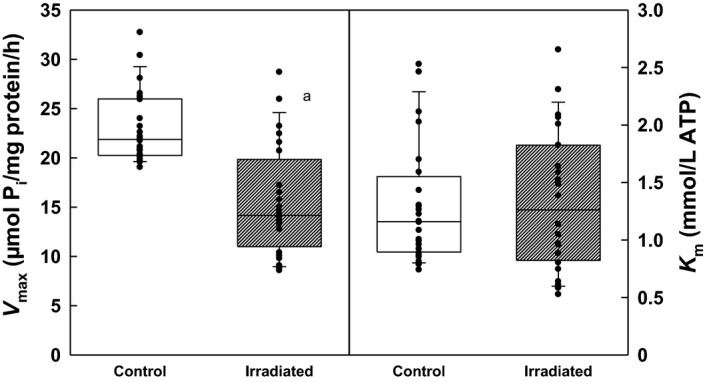
Kinetic parameters of renal Na,K‐ATPase during activation with ATP in control and irradiated (25 Gy) Wistar rats. Statistical significance: a ∼ *P* < 0.001.

Irradiation reduced the renal Na,K‐ATPase activity throughout the investigated concentration range of substrate as compared with controls. During the whole ATP concentration range, the decrease in activity represented 32–34% (Fig. 5). These changes in activities were reflected in statistically significant decrease in *V*_max_ by 33% with no significant alterations of *K*_m_ in irradiated rats (Fig. 6).

**Figure 7:**
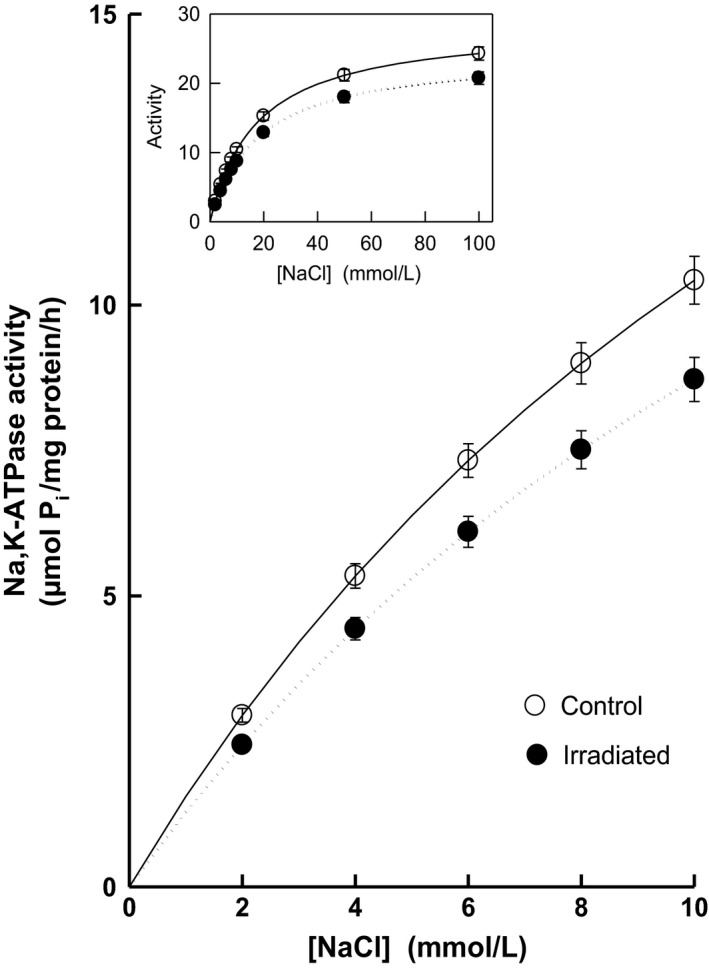
Activation of renal Na,K‐ATPase by low concentrations of NaCl in control and irradiated (25 Gy) Wistar rats. Insert: Activation of the enzyme in the whole investigated concentration range of NaCl.

**Figure 8:**
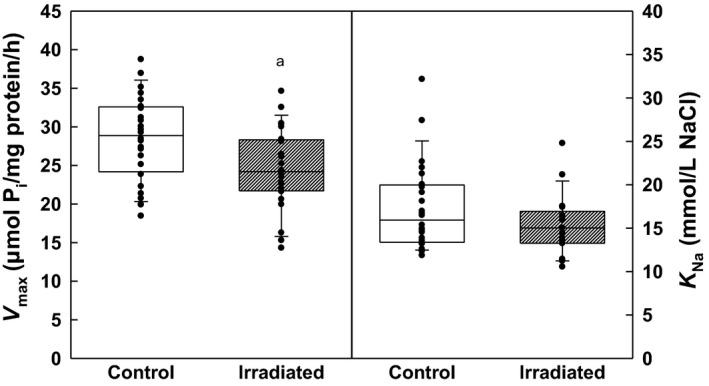
Kinetic parameters of renal Na,K‐ATPase during activation with NaCl in control and irradiated (25 Gy) Wistar rats. Statistical significance: a ∼ *P* < 0.05.

Comparing the response of Na,K‐ATPase to increasing concentrations of the cofactor Na^+^ we observed in the irradiated group again a considerable impairment of the enzymatic activity as witnessed by 14–17% decrease in the enzyme activity throughout the concentration range of NaCl (Fig. 7). These changes in activities were manifested in statistically significant decrease in *V*_max_ by 14% with no significant alterations of *K*_Na_ in irradiated rats (Fig. 8).

### Western blot analysis of renal Na,K‐ATPase

**Figure 9:**
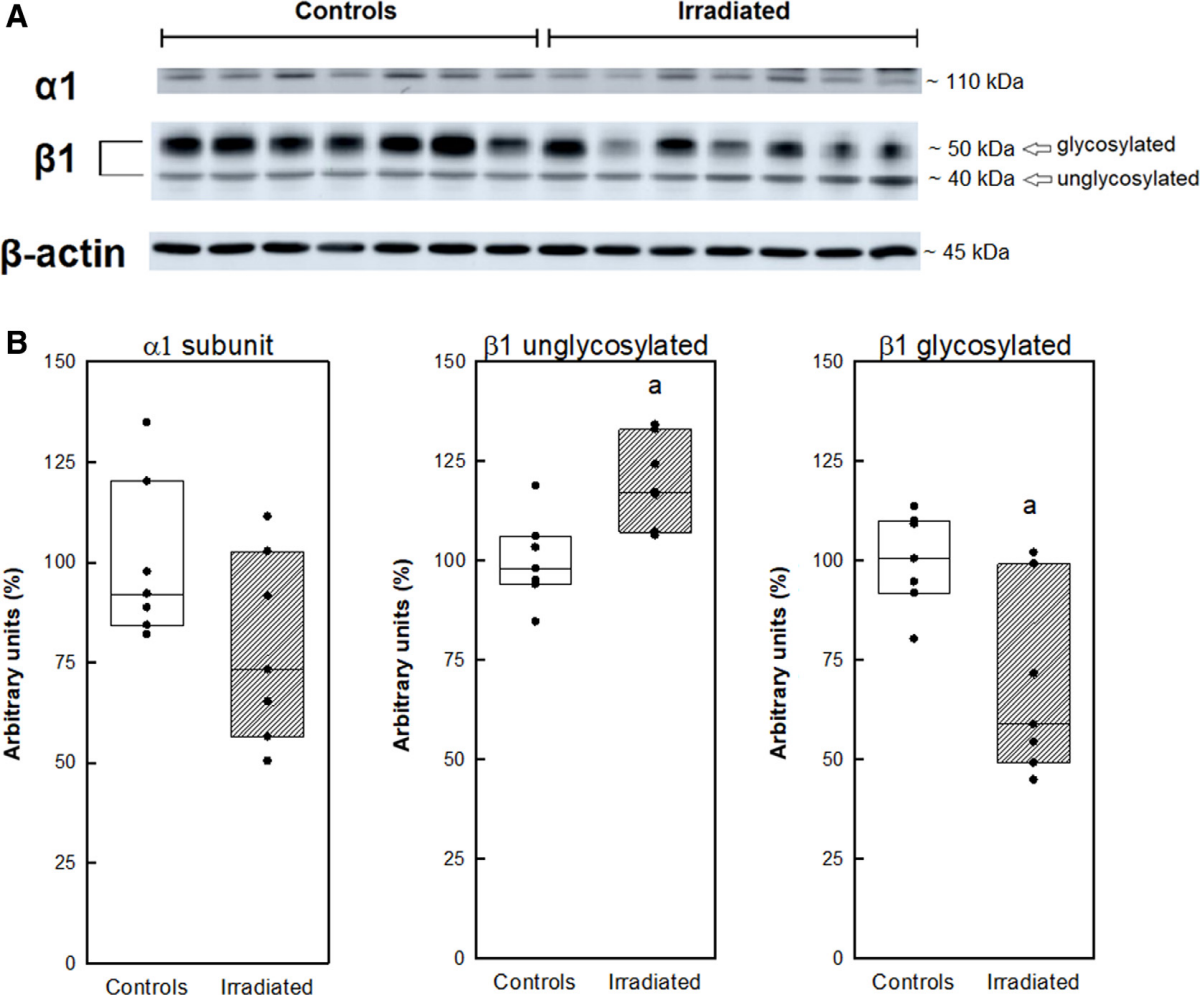
Representative immunoblot analysis of *α*1 and *β*1 subunits of Na,K‐ATPase in kidneys of control and irradiated (25 Gy) Wistar rats (A). Relative abundance of *α*1 and *β*1 subunits of Na,K‐ATPase. Statistical significance: a ∼ *P* < 0.01 as compared to the control group (B).

Analysis of Na,K‐ATPase subunits by western blot (Fig. 9) showed a tendency to lower presence of *α*1 subunit in irradiated rats in comparison with control animals. Evaluation of *β*1 subunit expression revealed distinct results for glycosylated and unglycosylated forms of this subunit. On the one side, the presence of glycosylated form was lower by more than 30%, on the other side the expression of the unglycosylated form was higher by 20% in the renal tissue of irradiated rats when compared with controls (Fig. 9). However, it should be mentioned that the cumulative presence of unglycosylated and glycosylated forms of *β*1 subunit remained unchanged after irradiation.

## Discussion

It is known that various anticancer therapies including radiotherapy can lead to cardiovascular complications and their severity depends on many factors including the site of action, the applied dose, and the method of administration. Cardiotoxicity can occur immediately upon administration of the anticancer therapy or it may have a delayed onset (months or years after the treatment) (for review see Rygiel 2017). Experimental studies oriented to local irradiation of the heart/thorax of rats using doses of 15–30 Gy resulted in cardiotoxic effects during the time interval of 60–240 days after application (Wondergem et al. 1991). Our previous study concerning the cardiac Na,K‐ATPase showed deterioration of the enzyme function 6 weeks after 25 Gy irradiation via the mechanism of lowered ability to bind sodium as well as the lowered number of active enzyme molecules (Mézešová et al. 2014). In the present study, we focused our attention to possible remote effect of irradiation, i.e. if irradiation of the body in one locality can affect another, unexposed part of the body. For maintenance of sodium homeostasis throughout the organism, the appropriate functionality of Na,K‐ATPase in renal tissue is very important. Therefore, we tried to bring new information concerning the effect of irradiation of rats in the mediastinal area on the properties of Na,K‐ATPase in kidneys.

### Plasma protein concentration and biochemical analysis of oxidative status

We were able to detect lower plasmatic concentration of proteins as a consequence of mediastinal irradiation. Hypoproteinemia post radiotherapy was already observed in other animal experiments (Nandchahal et al. 1990; El‐Gazzar et al. 2016). Lower concentrations of plasma proteins usually reflect low albumin concentrations. Preoperative radiation for retroperitoneal sarcoma was associated with more frequent hypoalbuminemia in humans (Bartlett et al. 2014). The observed hypoproteinemia after irradiation may be the consequence of liver injury, as liver is the major contributor of plasma proteins. It was shown that radiation‐induced liver disease involved hypoalbuminemia in patients who underwent radiotherapy for hepatocellular carcinoma (Furuse et al. 2005). The measurement of protein concentration postradiation is significant in clinics, since hypoalbuminemia was recognized as an independent predictor of poor outcome of selected malignities (Wang et al. 2009; Kang et al. 2016).

Together with alteration of protein concentration, negative impact of irradiation was also reflected in antioxidant status and oxidative stress in plasma. Lowered antioxidant status measured in the plasma after irradiation (TAC and FRAP parameters) is in concordance with human (Khalil Arjmandi et al. 2016) as well as animal (Manda et al. 2007) studies. Together with worsening antioxidant defense, we observed increased AGE formation that may be a consequence of increased production of reactive oxygen species (ROS), a potential cause of acute and chronic toxicity from irradiation (Alikhani et al. 2007; Yang et al. 2016).

### Histology of kidneys

The seriousness of pathophysiological complications in our experiment was emphasized by lower body weight gain and also by mortality amounting 17% in the group of irradiated animals. The lower size of glomeruli could be related with the decreased size of kidneys as part of the whole reduction in body weight of the irradiated animals. Similar correlation has been described also in humans (Nyengaard and Bendtsen 1992). The functionality of kidneys seems to be altered as indicated by our histological observations showing decrease in the size of kidney glomeruli together with increased optical density of blue‐stained tiny grains representing mitochondria in the tissue of irradiated animals. Surprisingly, our histological data providing information about increased density of mitochondria in the cytoplasm of epithelial cells of proximal tubules in surviving animals may be of physiological relevance. In this context, it is important to mention that only 83% of irradiated animals survived. So it might be suggested that altered presence of mitochondria in kidneys of irradiated rats may represent a possible compensatory effect to irradiation. It is known that renal epithelial cells contain high densities of mitochondria necessary to produce sufficient ATP for the active transport of Na^+^ ions. Approximately 90% of oxygen extracted by mitochondria in the kidney is used for Na^+^ reabsorption in the nephron (Welch 2006). From this point of view the alteration of renal mitochondria may be followed by alteration of functionality of Na,K‐ATPase and extrusion of superfluous sodium out from cells.

### Na,K‐ATPase discussion

**Figure 10:**
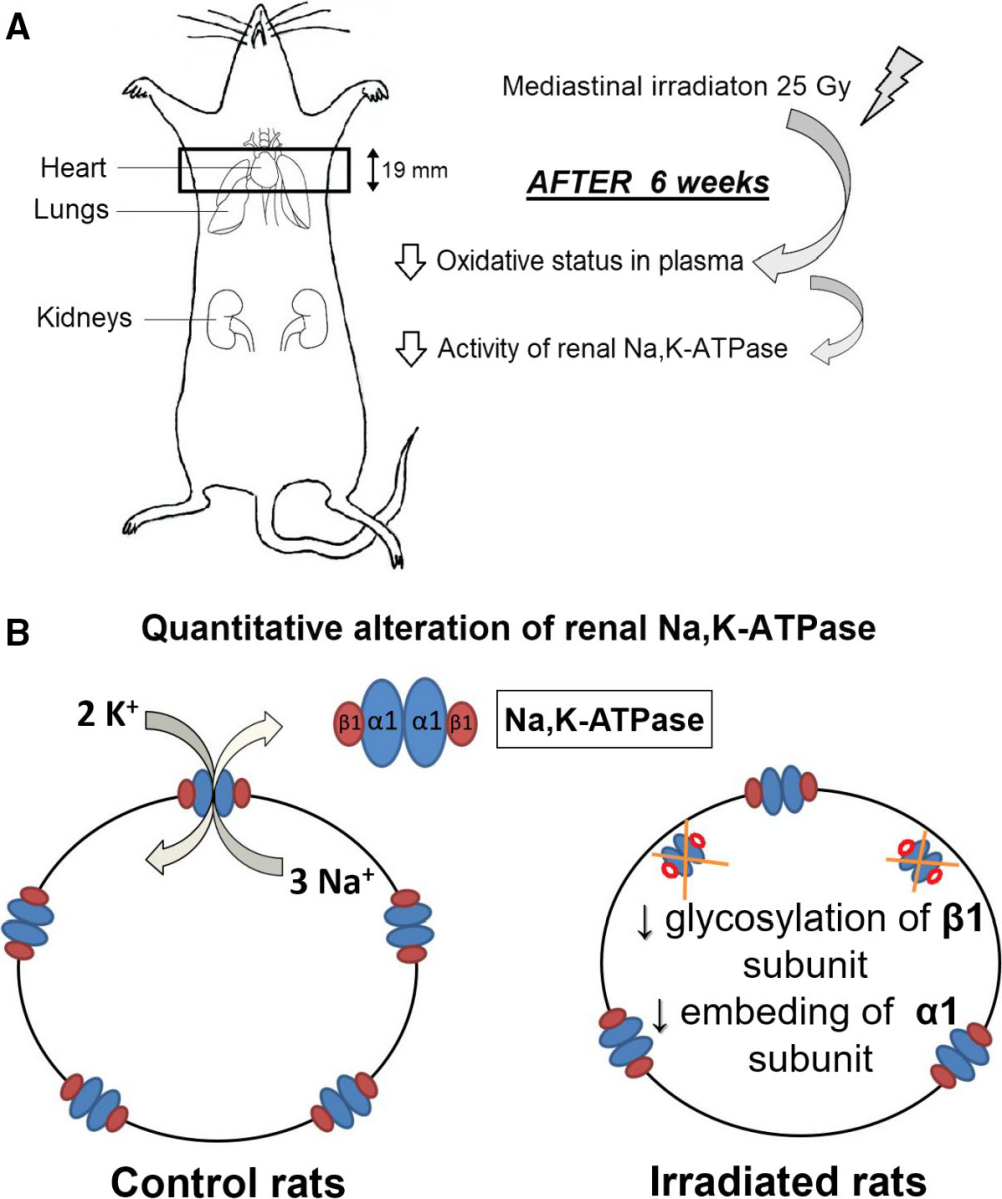
Schematic presentation of factors influencing the remote effect 6 weeks after mediastinal irradiation on the renal Na,K‐ATPase. The main supposed mechanisms involved in the remote effects of irradiation (A). Proposed mechanism of alteration of Na,K‐ATPase activity in kidneys of irradiated rats (B).

Even if, plasma creatinine and urea, the commonly used markers of renal function did not indicate significant functional alterations of kidney, the functionality of renal Na,K‐ATPase was influenced by irradiation. Evaluation of our enzyme kinetic data provides two basic information about the mechanism of Na,K‐ATPase alterations as a consequence of irradiation. Concerning the qualitative properties, the ability of the enzyme to bind the substrate ATP, as well as Na^+^ was not affected, as shown by unaltered values of *K*_m_ and *K*_Na_. Concerning the quantitative alterations, our study revealed new interesting findings. Based on the data of our present study the deterioration of the renal Na,K‐ATPase may be hypothesized as a remote effect of irradiation of rats in mediastinal region (Fig. 10). The decreased Na,K‐ATPase activity in irradiated rats is probably caused by lower number of active enzyme molecules as indicated by decreased value of *V*_max_ when compared to the control group. This difference was significant in both types of kinetic studies, it means during the activation of the enzyme with increasing concentration of both ‐ substrate ATP and cofactor sodium. So the Na,K‐ATPase extrudes less effectively the superfluous sodium out from cells in the kidneys of irradiated rats. The above hypothesis based on enzyme kinetics is supported also by our western blot analysis results documenting a tendency of lower expression of the *α*1 subunit in the Na,K‐ATPase in the renal tissue of irradiated rats. This subunit is generally recognized as the catalytic subunit of the Na,K‐ATPase, because it contains the binding sites for substrate ATP, as well as for cofactors sodium and potassium, thus securing the transmembrane transport of the above ions against their concentration gradients. The alterations in the expression of the glycososylated and unglycosylated forms of the *β*1seem to be very important. This subunit is responsible for correct embedding of the *α*‐subunit onto the surface membrane of the cell. The *β*‐subunit of renal Na,K‐ATPase is a sialoglycoprotein containing three potential N‐glycosylation sites (Miller and Farley 1988; Ataei and Wallick 1992). Previously it was documented that glycosylation of the Na,K‐ATPase *β*1 subunit is essential for the stable association of the pump with the adherens junctions and plays an important role in cell–cell contact formation in the kidney cells (Vagin et al. 2006). In addition, it was shown that inactivation of Na,K‐ATPase by deglycosylation is affected by interaction with surrounding lipids. It was documented that in the presence of dioleoylphosphatidylcholine, the deglycosylated enzyme was inactivated, whereas dioleoylphosphatidylserine protected the deglycosylated enzyme, and the Na,K‐ATPase activity was preserved (Cohen et al. 2005). So, the increased presence of unglycosylated *β*1 on the expense of the glycosylated *β*1 subunit may be the reason of worse incorporation of the catalytic *α*1 subunit into the plasmalemma resulting in decreased number of active Na,K‐ATPase molecules in the renal tissue of irradiated rats. The above deterioration of the Na,K‐ATPase function in kidney as a consequence of remote irradiation in the mediastinal area may be mediated by reactive oxygen species as indicated by worsened oxidative status in the plasma of irradiated rats. This proposal is in agreement with previous observations that oxidative stress in other pathological situations like hypertension or diabetes reduced electrolyte transport efficiency (Welch et al. 2001) and caused mitochondrial uncoupling (Friederich et al. 2009). Reduction of tubular electrolyte transport efficiency was hypothesized as a result of an interplay of several different mechanisms, including altered paracellular electrolyte permeability, direct effects on Na,K‐ATPase, and the shift of Na^+^ transport to less‐efficient nephron segments (Hansell et al. 2013). The increased presence of unglycosylated *β*1 subunit in the renal tissue of irradiated rats may be a consequence of deterioration of the nuclei forasmuch as in the kidneys of mice subjected to radiation dose 13–15 Gy, tubular cells with abnormally large nuclei were observed. For this abnormality it was suggested that it may belong to steps involved in cell loss in proximal tubules after irradiation (Otsuka et al. 1988).

### Comparison of cardiac and renal Na,K‐ATPase

The difference in the response of Na,K‐ATPase depending on the distance from the site of irradiation seems to be an interesting fact. In the heart, which was subjected directly to irradiation, the enzyme showed decrease in the number of active molecules together with lowered ability to bind the energy substrate ATP and also sodium ions (Mézešová et al. 2014). On the other hand, the present study showed that in kidneys which were during the irradiation procedure protected by lead shield, the enzyme revealed lowered presence of active molecules without significant effects on the ability to bind ATP and sodium.

## Conclusion

It may be summarized that irradiation by 25 Gy in the mediastinal area of rats induced remote deteriorating effects in other parts of the body despite their protection by lead shield. In the mediation of this process, probably oxygen radicals are responsible as documented by worsened oxidative status of blood plasma. The functionality of the main consumer of the intracellular ATP in renal cells, the Na,K‐ATPase, was also disturbed as documented by lowered number of active molecules as a consequence of worse incorporation of the catalytic alfa subunit into the plasmalemmal membrane. This system localized in the surface membrane of cells may represent one of the first systems injured by irradiation.

## Conflict of Interest

No conflicts of interest, financial or otherwise, as declared by the authors.
